# Blue Hg Dilemma and implications for Nature-based Solution and One Health Approach

**DOI:** 10.1016/j.eehl.2025.100152

**Published:** 2025-05-06

**Authors:** Zhijia Ci

**Affiliations:** Guangdong Provincial Key Laboratory of Marine Resources and Coastal Engineering, School of Marine Sciences, Sun Yat-sen University, Zhuhai 519082, China

The global community is seeking novel approaches to address major global challenges such as climate change, sea-level rise, biodiversity loss, and public health. Nature-based Solutions (NbS) and the One Health Approach (OHA) are two promising pathways. NbS are actions to leverage the power of healthy ecosystems to protect people and nature and safeguard a stable and sustainable future [1]. OHA is a pathway to safeguard water, food, and nutrient security and enhance our ability to tackle global health threats by emphasizing the interdependence between people, plants, animals, and their shared environment [2]. Obviously, these two concepts share identical core values, that is, promoting ecosystem sustainability, mitigating global change, and benefiting nature and people in an integrative way.

As a subset of NbS, Blue Carbon Strategies (BCSs)—protecting and expanding Blue Carbon ecosystems (seagrass meadow, salt marsh, and mangrove forest)—can enhance carbon sequestration and storage in coastal environments [3]. BCEs are now recognized as a promising approach to stabilize atmospheric CO_2_ levels and deliver co-benefits, including but not limited to increasing biodiversity, improving water quality, and buffering the impacts of sea-level rise and storms, which ultimately benefit both nature and people [3,4].

Recently, we reported the close linkage and co-accumulation of climate-friendly organic carbon and neurotoxic mercury (Hg) in global Blue Carbon ecosystems and estimated that over 95% of the Hg stock in global Blue Carbon ecosystems (termed Blue Hg) is stored in vegetated coastal ecosystems, due to co-accumulation of Hg during the assimilation of atmospheric CO_2_ into organic carbon via photosynthesis [5]. These findings indicate that the implementation of BCSs is very likely to increase Blue Hg stocks. Since the coastal sediments are the hotspots of the Hg biogeochemical cycle and coastal fisheries are the primary exposure pathway of the general population to Hg, this increase in Blue Hg stock may promote the production of methylmercury (the most toxic and bioaccumulative form of Hg) and its bioaccumulation in coastal food webs [6,7]. Ultimately, this could compromise ecosystem integrity, pose risks to human health, and hinder progress toward the United Nations Sustainable Development Goals [8,9]. Therefore, BCSs to mitigate and adapt to climate change and protect biodiversity and fisheries could inadvertently lead to an increase in the risk of Hg (termed Blue Hg Dilemma) [5], which is contrary to the core value of NbS and OHA.

Blue Hg Dilemma is a typical example showing the complexity of applying innovative approaches, like NbS and OHA, to address global challenges and achieve sustainable development in the context of a rapidly changing world. We emphasize interdisciplinary collaboration to comprehensively assess the effects of these approaches on nature and people, and make sound decisions that can simultaneously promote climate change mitigation, ecosystem and biodiversity conservation, and human well-being enhancement for present and future generations [10].

## CRediT authorship contribution statement

**Zhijia Ci:** Writing – review & editing, Writing – original draft, Project administration, Methodology, Investigation, Funding acquisition, Conceptualization.

## Declaration of competing interest

The author declares no competing interest.

## References

[1] International Union for Conservation of Nature (IUCN) Nature-based Solutions. https://iucn.org/our-work/nature-based-solutions.

[2] World Health Organization (WHO) One health. https://www.who.int/health-topics/one-health#tab=tab_1.

[3] Duarte C.M., Losada I.J., Hendriks I.E., Mazarrasa I., Marbà N. (2013). The role of coastal plant communities for climate change mitigation and adaptation. Nat. Clim. Change.

[4] Macreadie P.I., Costa M.D.P., Atwood T.B., Friess D.A. (2021). Blue carbon as a natural climate solution. Nat. Rev. Earth Environ..

[5] Ci Z., Shen W., Chen B., Li Y., Yin Y., Zhang X., Cai Y. (2024). Potential increase of neurotoxic mercury risk in global Blue carbon nature-based Solutions. Nat. Sustain..

[6] Ci Z., Yin Y., Shen W., Chen B. (2023). Non-conservative mixing behaviors of mercury in subterranean estuary: coupling effect of hydrological and biogeochemical processes and implications for rapidly changing world. Water Res..

[7] Lei P., Zhong H., Duan D., Pan K. (2019). A review on mercury biogeochemistry in mangrove sediments: hotspots of methylmercury production?. Sci. Total Environ..

[8] Schartup A.T., Thackray C.P., Qureshi A., Dassuncao C., Gillespie K., Hanke A., Sunderland E.M. (2019). Climate change and overfishing increase neurotoxicant in marine predators. Nature.

[9] Tseng C.-M., Ang S.-J., Chen Y.-S., Shiao J.-C., Lamborg C.H., He X., Reinfelder J.R. (2021). Bluefin tuna reveal global patterns of mercury pollution and bioavailability in the World's oceans. Proc. Natl. Acad. Sci. U.S.A..

[10] Ci Z., Shen W., Chen B., Li Y., Yin Y., Zhang X., Cai Y. (2024). Mercury risk in Blue carbon ecosystems. Nat. Sustain..

